# From Orphan Phage to a Proposed New Family–The Diversity of N4-Like Viruses

**DOI:** 10.3390/antibiotics9100663

**Published:** 2020-09-30

**Authors:** Johannes Wittmann, Dann Turner, Andrew D. Millard, Padmanabhan Mahadevan, Andrew M. Kropinski, Evelien M. Adriaenssens

**Affiliations:** 1 Leibniz Institute DSMZ–German Collection of Microorganisms and Cell Cultures, 38124 Braunschweig, Germany; 2 Department of Applied Sciences, University of the West of England, Bristol BS16 1QY, UK; dann2.turner@uwe.ac.uk; 3 Department of Genetics and Genome Biology, University of Leicester, Leicester LE1 7RH UK; adm39@le.ac.uk; 4 Department of Biology, University of Tampa, Tampa, FL 33606, USA; pmahadevan1@gmail.com; 5 Department of Food Science, University of Guelph, Guelph, ON N1G 2W1, Canada; phage.canada@gmail.com; 6 Department of Pathobiology, University of Guelph, Guelph, ON N1G 2W1, Canada; 7 Quadram Institute Bioscience, Norwich Research Park, Norwich NR4 7UQ, UK; evelien.adriaenssens@quadram.ac.uk

**Keywords:** N4, *Schitoviridae*, virus taxonomy, ICTV, bacteriophages, bacterial viruses

## Abstract

Escherichia phage N4 was isolated in 1966 in Italy and has remained a genomic orphan for a long time. It encodes an extremely large virion-associated RNA polymerase unique for bacterial viruses that became characteristic for this group. In recent years, due to new and relatively inexpensive sequencing techniques the number of publicly available phage genome sequences expanded rapidly. This revealed new members of the *N*4-like phage group, from 33 members in 2015 to 115 N4-like viruses in 2020. Using new technologies and methods for classification, the Bacterial and Archaeal Viruses Subcommittee of the International Committee on Taxonomy of Viruses (ICTV) has moved the classification and taxonomy of bacterial viruses from mere morphological approaches to genomic and proteomic methods. The analysis of 115 N4-like genomes resulted in a huge reassessment of this group and the proposal of a new family “*Schitoviridae*”, including eight subfamilies and numerous new genera.

## 1. Introduction

**Figure 1:**
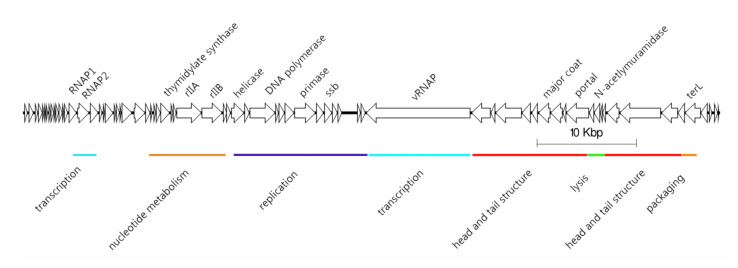
Genome structure of Escherichia phage N4 (70,153 bp) visualized by EasyFig [7].

Escherichia phage N4 is a virulent phage that was originally isolated by Gian Carlo Schito from sewers in Genoa (Italy) in 1966 [1]. TEM analysis revealed a 70-nm-diameter capsid and a short tail, the characteristic features of a podovirus. Its genome consists of double-stranded DNA and has a length of 70,153 bp with about 400 bp direct repeats and short 3′-noncohesive extensions [2]. Analysis of the *N*4 genome and its replication revealed unique characteristics. Apart from a phenomenon called lysis-inhibition [3] that causes delayed lysis and a subsequently increased burst size upon infection, further analysis revealed a gene for a large virion-associated RNA polymerase (vRNAP) that became a characteristic for N4-like genomes and the use of in total three DNA-dependent RNA polymerases for transcription that were subject to different scientific questions and thus were intensively studied (Figure 1) [4]. The vRNAP is injected into the host cell along with DNA [5]. N4 genome is transcribed in three different temporal stages [6].

From a taxonomic perspective, phage N4 has a long history; from the first proposal in 1987 to establish a species Escherichia phage N4 [8] and its subsequent renaming to *Escherichia virus N4* in 2015, it has persisted as a genomic orphan. At the time of writing (Master Species List #35, ratified March 2020), its genus is now called *Enquatrovirus* and consists of only one species, representing four isolates. 

Since 2015, following a comprehensive analysis of at that time 33 N4-like genomes [9], the number of publicly available N4-like phage genomes has nearly tripled [10]. The last report of the Bacterial and Archaeal Viruses Subcommittee [11] presented the new taxonomic classifications and reassessments that were achieved in 2018 and 2019 and listed a new order (*Tubulavirales*), ten new families, 22 new sub-families, 424 new genera and 964 new species, which still represent only a fraction of the genomes currently available. However, it has to be taken into account that ICTV does not classify viral strains or variants, i.e., those phage isolates with genomes that show ≥95% DNA sequence identity with an exemplar isolate of a species [12]. With regard to N4-like viruses, i.e., viruses encoding the vRNAP, only a rather small number of those have been officially classified by the ICTV so far. Currently, they are classified in 10 genera (*Baltimorevirus*, *Enquatrovirus*, *Gamaleyavirus*, *Ithacavirus*, *Johnsonvirus*, *Jwalphavirus*, *Litunavirus*, *Luzseptimavirus*, *Mukerjeevirus* and *Shizishanvirus*). This study provides further insight into the diversity and taxonomy of *N4-like viruses* using different approaches like genome-based phylogeny for deeper classification.

## 2. Results

### 2.1. Description of N4-Like Viruses

We downloaded 115 genomes from the NCBI databases (INSDC) [13,14] related to the N4-like group of viruses (Table 1). All N4-like phages and members of the proposed new family share the following characteristics:

- Podovirus morphology
- Genome size of 59–80 kb
- Linear genome with defined ends (terminal repeats expected)
- Presence of three RNA polymerase genes, including a large (~3500 aa) virion-associated RNA polymerase (vRNAP)

So far, only N4-like phages infectious for Gram-negative host bacteria belonging to the α-proteobacteria such as *Roseobacter* [21], β-proteobacteria such as *Achromobacter* [3] and γ-proteobacteria such as *Pseudomonas* [43] from different habitats have been described. From the morphological perspective, N4-like phages show characteristic features of podoviruses, capsid sizes range from 50 [35] to 85 nm [63] with short non-contractile tails.

### 2.2. Proposal of a New Family

**Figure 2:**
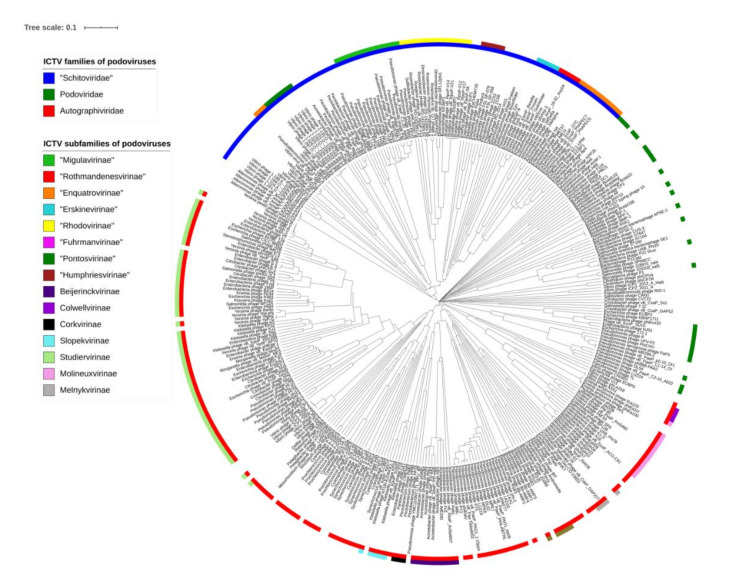
ViPTree analysis of N4-like viruses with related podoviruses. Results were visualized with iTol. Viruses were assigned and marked according to the official ICTV classification with the outer and inner rings representing classification at the subfamily and family level, respectively. Non-marked viruses have not been classified yet.

**Figure 3:**
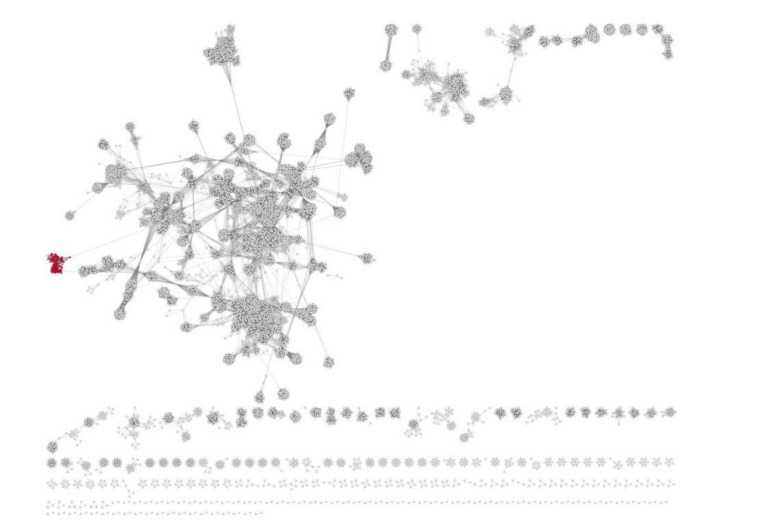
VConTACT2 network analysis. Members of the proposed “*Schitoviridae*” family are marked in red.

To analyze the similarities or relationship, respectively, between N4-like viruses and other podoviruses, we used ViPTree (https://www.genome.jp/viptree/; [66]) which is originally based on the Phage Proteomic Tree [67]. The results showed that the group of N4-like is clearly monophyletic and forms a distinct clade (Figure 2). The distinct clustering of the newly proposed family was confirmed with a gene-sharing network analysis using vConTACT2 (Figure 3), where the N4-like viruses cluster clearly separates from all other dsDNA bacterial viruses. In fact, the deep branch lengths in the ViPTree and limited connectedness in the gene-sharing network show that there are no unifying genomic features among all members of the *Podoviridae* to justify the current membership.

Panproteome analysis revealed that seventeen N4-like proteins are conserved in this proposed family of phages: RNAP 1 (EPNV4_gp15), RNAP 2 (EPNV4_gp16), vRNAP (EPNV4_gp50), EPNV4_gp24, EPNV4_gp25, DNA polymerase (EPNV4_gp39), EPNV4_gp42, DNA primase (EPNV4_gp43), EPNV4_gp44, EPNV4_gp52, EPNV4_gp54, major capsid protein (EPNV4_gp56), tape measure protein (EPNV4_gp57), portal protein (EPNV4_gp59), EPNV4_gp67, large terminase subunit (EPNV4_gp68), and EPNV4_gp69 (Table 2).

Based on the different analyses, we propose a new family “*Schitoviridae*” in honor of Gian Carlo Schito who isolated Escherichia phage N4, the first isolated species of this group.

### 2.3. Proposal of New Subfamilies and Genera

Results of an all-by-all pairwise nucleotide identity analysis or intergenomic similarity analysis with VIRIDIC gave strong evidence for the proposal of eight new subfamilies and 30 genera which were confirmed by phylogenetic analysis of the terminase large subunit and vRNA polymerase genes, i.e., all proposed taxa are monophyletic in these marker gene trees (Supplementary Figures S1 and S2). In line with previously established taxa, we used 95% and 70% nucleotide sequence identity over the length of the genome as species and genus demarcation criteria, respectively [11,12,68,69]. At the subfamily level, members of the same subfamily share at least 40% intergenomic distance as calculated with VIRIDIC, with members of different subfamilies sharing little to no nucleotide identity [70].

The proposed subfamily “*Migulavirinae*” consists of two previously ratified genera (eight species), *Litunavirus* and *Luzseptimavirus*, representing phages with *Pseudomonas aeruginosa* as their host. The subfamily “*Enquatrovirinae*” contains three genera (14 species), *Gamaleyavirus*, *Enquatrovirus* and the newly proposed genus “*Kaypoctavirus*” and includes phages infecting members of the *Enterobacteriaceae* like *E. coli*, *Shigella boydii* or *Klebsiella pneumoniae*. N4-like viruses infecting *Achromobacter xylosoxidans* were grouped into four genera (eight species) in the proposed subfamily “*Rothmandenesvirinae*” in honour of Lucia Rothman-Denes, who worked on N4 and its RNA polymerases. The subfamily “*Erskinevirinae*” was named after John M. Erskine who in the early 1970s was one of the first people to isolate phages against *Erwinia*. It consists of two genera, “*Yonginvirus*” and *Johnsonvirus*, with three species and represents most of the N4-like viruses against *Erwinia*. The relatively large subfamily “*Rhodovirinae*” consists of seven genera, “*Aorunvirus*”, “*Raunefjordvirus*”, “*Aoquinvirus*”, “*Pomeroyivirus*”, “*Sanyabayvirus*”, “*Plymouthvirus*” and *Baltimorevirus*, and contains aquatic viruses infecting members of the *Rhodobacteraceae*. Two further proposed subfamilies (five genera), “*Fuhrmanvirinae*” (named after American oceanographer and marine biologist Jed Alan Fuhrman) and “*Pontosvirinae*”, mainly consist of phages against marine *Vibrio* species. The “*Humphriesvirinae*” subfamily in honour of James C. Humphries (1914–1992), who was the first to isolate a Klebsiella phage, comprises five genera with viruses infecting different genera of the *Enterobacteriaceae* like *Escherichia*, *Klebsiella* or *Salmonella*.

## 3. Discussion

The constantly rising number of sequences provides the scientific community with valuable data to work with to answer various scientific questions. However, the taxonomic classification of phage genomes has not kept pace which has led to the presence of large numbers of unclassified genomes in the INSDC. While the ICTV makes a huge effort to manage this problem and improvements have been made on the genus and subfamily level (2019: 103 proposals, 2020: 188 proposals submitted [68]), it is clear that at the family level that concerted efforts, both by the ICTV and the wider community of phage biologists are required to address the issue of family-level classification. The creation of the family *Herelleviridae* from the subfamily *Spounavirinae* and related phages [69], provided the blueprint for the creation of new families of tailed phages, and the start to the dismantling of the morphology-based families *Myoviridae, Siphoviridae* and *Podoviridae*. Following from that example, we used some of the methods trialed and tested for the creation of a new family (Phage Proteomic Tree, vConTACT2) and the delineation of its internal structure (genome-distance comparisons, phylogenetic analysis of signature genes) to define the new family “*Schitoviridae*” of N4-like phages, to be removed from the family *Podoviridae*.

## 4. Materials and Methods

### 4.1. vConTACT2 Analysis

To create the gene-sharing network, a total of 16,050 phage contigs (http://millardlab.org/) [71] were reannotated using Prodigal v2.6.3 and clustered using vConTACT.2.0 [72] and the ProkaryoticViralRefSeq database v94. The resulting network was visualised and annotated using Cytoscape v3.8.0.

### 4.2. Panproteome Analysis

To identify conserved proteins present in bacteriophages comprising the proposed family, all genomes were reannotated using Prokka v1.14.5 [73] and predicted CDS mapped against the VOG hmm database using hmmscan. GFF3 files or protein FASTA files were used as input for Proteinortho v6 [74] and PIRATE v1.0.4 [75], respectively.

For panproteome construction with PIRATE the settings used were 30 and 35% identity threshold, cdhit lowest percentage id of 95 and e-value for BLAST hit filtering of 1E-5. For Proteinortho, the search options were adjusted so that the minimum percent identity and coverage of the best blast hits were 30% and 50%, respectively. All other parameters were left as default.

The CoreGenes5.0 webserver (https://coregenes.ngrok.io/) was used with the OrthoMCL option with E-value of 1e-5. CoreGenes5.0 uses the GET_HOMOLOGUES package to implement the ortholog clustering [76,77]. We considered signature genes to be gene products present in all members of the proposed family where there was consensus between two or more of the analyses.

### 4.3. VIRIDIC Analysis

The Bacterial and Archaeal Viruses Subcommittee uses nucleotide based sequence similarities as a crucial feature for taxonomic classification of viruses at the ranks of species and genus. We therefore employed the online tool VIRIDIC (Virus Intergenomic Distance Calculator, http://rhea.icbm.uni-oldenburg.de/VIRIDIC/) [70] for the calculation of pairwise intergenomic similarities amongst the phage genomes of this study. We have chosen 95% DNA sequence identity as the criterion for demarcation of species in genera. Each of the proposed species differs from the others with more than 5% at the DNA level. For the demarcation of genera and subfamilies, we have chosen 70% and 40% DNA sequence identity, respectively. Based on this analysis, new genera and subfamilies were identified (Supplementary Table S1).

## Figures and Tables

**Table 1:** List of N4-like genomes proposed to belong to the new family “*Schitoviridae*” available from INSDC databases.

Phage	Subfamily	Genus	Reference
Achromobacter phage JWAlpha	*“Rothmandenesvirinae”*	*Jwalphavirus*	[15]
Achromobacter phage JWDelta	*“Rothmandenesvirinae”*	*Jwalphavirus*	[15]
Achromobacter phage phiAxp–3	*“Rothmandenesvirinae”*	*“Dongdastvirus”*	[16]
Achromobacter phage vB_AxyP_19–32_Axy04	*“Rothmandenesvirinae”*	*“Dongdastvirus”*	[17]
Achromobacter phage vB_AxyP_19–32_Axy10	*“Rothmandenesvirinae”*	*“Pourcelvirus”*	[17]
Achromobacter phage vB_AxyP_19–32_Axy11	*“Rothmandenesvirinae”*	*“Pourcelvirus”*	[17]
Achromobacter phage vB_AxyP_19–32_Axy12	*“Rothmandenesvirinae”*	*“Dongdastvirus”*	[17]
Achromobacter phage vB_AxyP_19–32_Axy13	*“Rothmandenesvirinae”*	*“Inbricusvirus”*	[17]
Achromobacter phage vB_AxyP_19–32_Axy22	*“Rothmandenesvirinae”*	*“Pourcelvirus”*	[17]
Achromobacter phage vB_AxyP_19–32_Axy24	*“Rothmandenesvirinae”*	*“Dongdastvirus”*	[17]
Acinetobacter phage Presley		*“Presleyvirus”*	[18]
Acinetobacter phage VB_ApiP_XC38			[19]
Delftia phage RG–2014		*“Dendoorenvirus”*	[20]
Dinoroseobacter phage DFL12phi1	*“Rhodovirinae”*	*Baltimorevirus*	
Dinoroseobacter phage DS–1410Ws–06	*“Rhodovirinae”*	*“Sanyabayvirus”*	[21]
Dinoroseobacter phage vBDshPR2C	*“Rhodovirinae”*	*Baltimorevirus*	[22]
Enterobacter phage EcP1		*“Eceepunavirus”*	
Enterobacteria phage *N*4	*“Enquatrovirinae”*	*Enquatrovirus*	[3]
Erwinia phage Ea9–2	*“Erskinevirinae”*	*Johnsonvirus*	
Erwinia phage phiEaP–8	*“Erskinevirinae”*	*Yonginvirus*	[23]
Erwinia phage vB_EamP_Frozen	*“Erskinevirinae”*	*Johnsonvirus*	[24]
Erwinia phage vB_EamP_Gutmeister	*“Erskinevirinae”*	*Johnsonvirus*	[24]
Erwinia phage vB_EamP_Rexella	*“Erskinevirinae”*	*Johnsonvirus*	[24]
Erwinia phage vB_EamP–S6		*“Waedenswilvirus”*	[25]
Escherichia phage Bp4	*“Enquatrovirinae”*	*Gamaleyavirus*	[26]
Escherichia phage EC1–UPM	*“Enquatrovirinae”*	*Gamaleyavirus*	[27]
Escherichia phage ECBP1	*“Enquatrovirinae”*	*Gamaleyavirus*	[28]
Escherichia phage IME11	*“Enquatrovirinae”*	*Gamaleyavirus*	[29]
Escherichia phage OLB145	*“Enquatrovirinae”*	*Enquatrovirus*	
Escherichia phage PD38	*“Enquatrovirinae”*	*Gamaleyavirus*	
Escherichia phage PGN829.1	*“Enquatrovirinae”*	*Gamaleyavirus*	
Escherichia phage phi G17	*“Enquatrovirinae”*	*Gamaleyavirus*	[30]
Escherichia phage PMBT57	*“Enquatrovirinae”*	*Enquatrovirus*	
Escherichia phage Pollock	*“Humphriesvirinae”*	*“Pollockvirus”*	[31]
Escherichia phage St11Ph5	*“Enquatrovirinae”*	*Gamaleyavirus*	[32]
Escherichia phage vB_EcoP_3HA13	*“Enquatrovirinae”*	*Enquatrovirus*	
Escherichia phage vB_EcoP_G7C	*“Enquatrovirinae”*	*Gamaleyavirus*	[33]
Escherichia phage vB_EcoP_PhAPEC5	*“Enquatrovirinae”*	*Gamaleyavirus*	[34]
Escherichia phage vB_EcoP_PhAPEC7	*“Enquatrovirinae”*	*Gamaleyavirus*	[34]
Klebsiella phage KP8	*“Enquatrovirinae”*	*“Kaypoctavirus”*	[35]
Klebsiella phage KpCHEMY26	*“Humphriesvirinae”*	*“Pylasvirus”*	[36]
Klebsiella phage Pylas	*“Humphriesvirinae”*	*“Pylasvirus”*	[37]
Pectobacterium phage Nepra		*“Cbunavirus”*	
Pectobacterium phage phiA41		*“Cbunavirus”*	[38]
Pectobacterium phage vB_PatP_CB1		*“Cbunavirus”*	[39]
Pectobacterium phage vB_PatP_CB3		*“Cbunavirus”*	[39]
Pectobacterium phage vB_PatP_CB4		*“Cbunavirus”*	[39]
Pseudoalteromonas phage pYD6-A	*“Fuhrmanvirinae”*	*“Mazuvirus”*	
Pseudomonas phage 98PfluR60PP		*“Littlefixvirus”*	[40]
Pseudomonas phage DL64	*“Migulavirinae”*	*Litunavirus*	[41]
Pseudomonas phage inbricus	*“Rothmandenesvirinae”*	*“Inbricusvirus”*	
Pseudomonas phage KPP21	*“Migulavirinae”*	*Luzseptimavirus*	[42]
Pseudomonas phage LIT1	*“Migulavirinae”*	*Litunavirus*	[43]
Pseudomonas phage Littlefix		*“Littlefixvirus”*	
Pseudomonas phage LP14	*“Migulavirinae”*	*Litunavirus*	[44]
Pseudomonas phage LUZ7	*“Migulavirinae”*	*Luzseptimavirus*	[43]
Pseudomonas phage LY218	*“Migulavirinae”*	*Litunavirus*	
Pseudomonas phage Pa2	*“Migulavirinae”*	*Litunavirus*	
Pseudomonas phage PA26	*“Migulavirinae”*	*Litunavirus*	[45]
Pseudomonas phage PEV2	*“Migulavirinae”*	*Litunavirus*	[43]
Pseudomonas phage phCDa		*Shizishanvirus*	
Pseudomonas phage phi176	*“Migulavirinae”*	*Litunavirus*	[46]
Pseudomonas phage RWG	*“Migulavirinae”*	*Litunavirus*	[46]
Pseudomonas phage vB_Pae1396P-5	*“Migulavirinae”*	*Litunavirus*	
Pseudomonas phage vB_Pae575P-3	*“Migulavirinae”*	*Litunavirus*	
Pseudomonas phage vB_PaeP_C2–10_Ab09	*“Migulavirinae”*	*Litunavirus*	[47]
Pseudomonas phage vB_PaeP_DEV	*“Migulavirinae”*	*Litunavirus*	[48]
Pseudomonas phage vB_PaeP_MAG4	*“Migulavirinae”*	*Litunavirus*	[49]
Pseudomonas phage vB_PaeP_PYO2	*“Migulavirinae”*	*Litunavirus*	[48]
Pseudomonas phage YH30	*“Migulavirinae”*	*Litunavirus*	[50]
Pseudomonas phage YH6	*“Migulavirinae”*	*Litunavirus*	[51]
Pseudomonas phage ZC03		*“Zicotriavirus”*	[52]
Pseudomonas phage ZC08		*“Zicotriavirus”*	[52]
Pseudomonas phage Zuri		*“Zurivirus”*	
Roseobacter phage RD–1410W1–01	*“Rhodovirinae”*	*“Aoquinvirus”*	[21]
Roseobacter phage RD–1410Ws–07	*“Rhodovirinae”*	*“Sanyabayvirus”*	[21]
Roseovarius Plymouth podovirus 1	*“Rhodovirinae”*	*“Plymouthvirus”*	[53]
Roseovarius sp. 217 phage 1	*“Rhodovirinae”*	*“Plymouthvirus”*	[53]
Ruegeria phage vB_RpoP–V12	*“Rhodovirinae”*	*“Aorunvirus”*	
Ruegeria phage vB_RpoP–V13	*“Rhodovirinae”*	*“Pomeroyivirus”*	
Ruegeria phage vB_RpoP–V14	*“Rhodovirinae”*	*“Aorunvirus”*	
Ruegeria phage vB_RpoP–V17	*“Rhodovirinae”*	*“Aorunvirus”*	
Ruegeria phage vB_RpoP–V21	*“Rhodovirinae”*	*“Aorunvirus”*	
Salmonella phage FSL SP–058	*“Humphriesvirinae”*	*“Ithacavirus”*	[54]
Salmonella phage FSL SP–076	*“Humphriesvirinae”*	*“Ithacavirus”*	[54]
Shigella phage pSb–1	*“Enquatrovirinae”*	*Gamaleyavirus*	[55]
Silicibacter phage DSS3phi2	*“Rhodovirinae”*	*“Aorunvirus”*	[56]
Sinorhizobium phage ort11		*“Huelvavirus”*	[57]
Stenotrophomonas phage Pokken		*“Pokkenvirus”*	[58]
Sulfitobacter phage EE36phi1	*“Rhodovirinae”*	*“Aorunvirus”*	[56]
Sulfitobacter phage phiCB2047-B	*“Rhodovirinae”*	*“Raunefjordvirus”*	[59]
Vibrio phage 1.025.O._10N.222.46.B6	*“Pontosvirinae”*	*“Nahantvirus”*	
Vibrio phage 1.026.O._10N.222.49.C7	*“Pontosvirinae”*	*“Nahantvirus”*	
Vibrio phage 1.097.O._10N.286.49.B3	*“Pontosvirinae”*	*“Dorisvirus”*	
Vibrio phage 1.150.O._10N.222.46.A6	*“Pontosvirinae”*	*“Nahantvirus”*	
Vibrio phage 1.152.O._10N.222.46.E1	*“Pontosvirinae”*	*“Nahantvirus”*	
Vibrio phage 1.169.O._10N.261.52.B1		*Mukerjeevirus*	
Vibrio phage 1.188.A._10N.286.51.A6		*Mukerjeevirus*	
Vibrio phage 1.224.A._10N.261.48.B1		*Mukerjeevirus*	
Vibrio phage 1.261.O._10N.286.51.A7		*Mukerjeevirus*	
Vibrio phage 2.130.O._10N.222.46.C2	*“Pontosvirinae”*	*“Nahantvirus”*	
Vibrio phage JA–1		*“Pacinivirus”*	[60]
Vibrio phage JSF3		*“Pacinivirus”*	[61]
Vibrio phage phi 1		*“Pacinivirus”*	[62]
Vibrio phage phi50–12			
Vibrio phage pVa5	*“Pontosvirinae”*	*“Galateavirus”*	[63]
Vibrio phage pVco–5			[64]
Vibrio phage VBP32	*“Fuhrmanvirinae”*	*“Stoningtonvirus”*	
Vibrio phage VBP47	*“Fuhrmanvirinae”*	*“Stoningtonvirus”*	
Vibrio phage VCO139		*“Pacinivirus”*	[60]
Vibrio virus vB_VspP_SBP1			
Xanthomonas phage RiverRider		*“Riverridervirus”*	[65]
**From metagenomes**			
*Podoviridae* sp. isolate ctda_1			
*Podoviridae* sp. ctLUJ1			
*Siphoviridae* sp. isolate 355	*“Enquatrovirinae”*	*Gamaleyavirus*	

**Table 2:** Panproteome analysis of N4-like viruses using three different approaches.

#	N4_Product	N4 Locus Tag	N4 Protein Accession	PIRATE	Proteinortho_30	CoreGenes 5.0
1	RNAP 1	EPNV4_gp15	YP_950493.1	Y	Y	Y
2	RNAP 2	EPNV4_gp16	YP_950494.1	N	Y	Y
3	AAA+ ATPase	EPNV4_gp24	YP_950502.1	*	Y	Y
4	gp25	EPNV4_gp25	YP_950503.1	N	N	Y
5	DNA polymerase	EPNV4_gp39	YP_950517.1	Y	Y	Y
6	gp42	EPNV4_gp42	YP_950520.1	Y	Y	Y
7	DNA primase	EPNV4_gp43	YP_950521.1	Y	Y	Y
8	gp44	EPNV4_gp44	YP_950522.1	Y	Y	Y
9	vRNAP	EPNV4_gp50	YP_950528.1	N	N	Y
10	16.5 kDa protein	EPNV4_gp52	YP_950530.1	Y	Y	Y
11	gp54	EPNV4_gp54	YP_950532.1	N	N	Y
12	Major capsid protein	EPNV4_gp56	YP_950534.1	Y	Y	Y
13	gp57 (tape measure)	EPNV4_gp57	YP_950535.1	N	*	Y
14	94 kDa protein (portal vertex protein)	EPNV4_gp59	YP_950537.1	Y	Y	Y
15	30 kDa protein	EPNV4_gp67	YP_950545.1	N	N	Y
16	Terminase, large subunit	EPNV4_gp68	YP_950546.1	Y	Y	Y
17	gp69	EPNV4_gp69	YP_950547.1	Y	N	Y

^*^ Indicates presence in 113/114 genomes.

## Supplementary Materials

The following are available online at https://www.mdpi.com/2079-6382/9/10/663/s1, Figure S1: Phylogenetic analysis using the terminase protein sequences of *N*4-like phages, respectively. The amino acid sequences were compared using MUSCLE with MEGA7 [78]. The tree was constructed using the maximum likelihood algorithm. The percentages of replicate trees were assessed with the bootstrap test (100).; Figure S2: Phylogenetic analysis using the vRNA polymerase protein sequences of *N*4-like phages, respectively. The amino acid sequences were compared using MUSCLE with MEGA7 [78]. The tree was constructed using the maximum likelihood algorithm. The percentages of replicate trees were assessed with the bootstrap test (100); Figure S3: ViPTree analysis of *N*4-like viruses with related podoviruses, Table S1: VIRIDIC analysis of *N*4-like phages.
