# Supplementary material for: From Orphan Phage to a Proposed New Family–The Diversity of N4-Like Viruses
Source: Antibiotics (Basel). 2020 Sep 30;9(10):663. doi: 10.3390/antibiotics9100663 (PMC7650795; doi:10.3390/antibiotics9100663)
Supplement: Supplementary file 1 [file antibiotics-09-00663-s001.zip › Supplementary files/Figure S3.pdf]

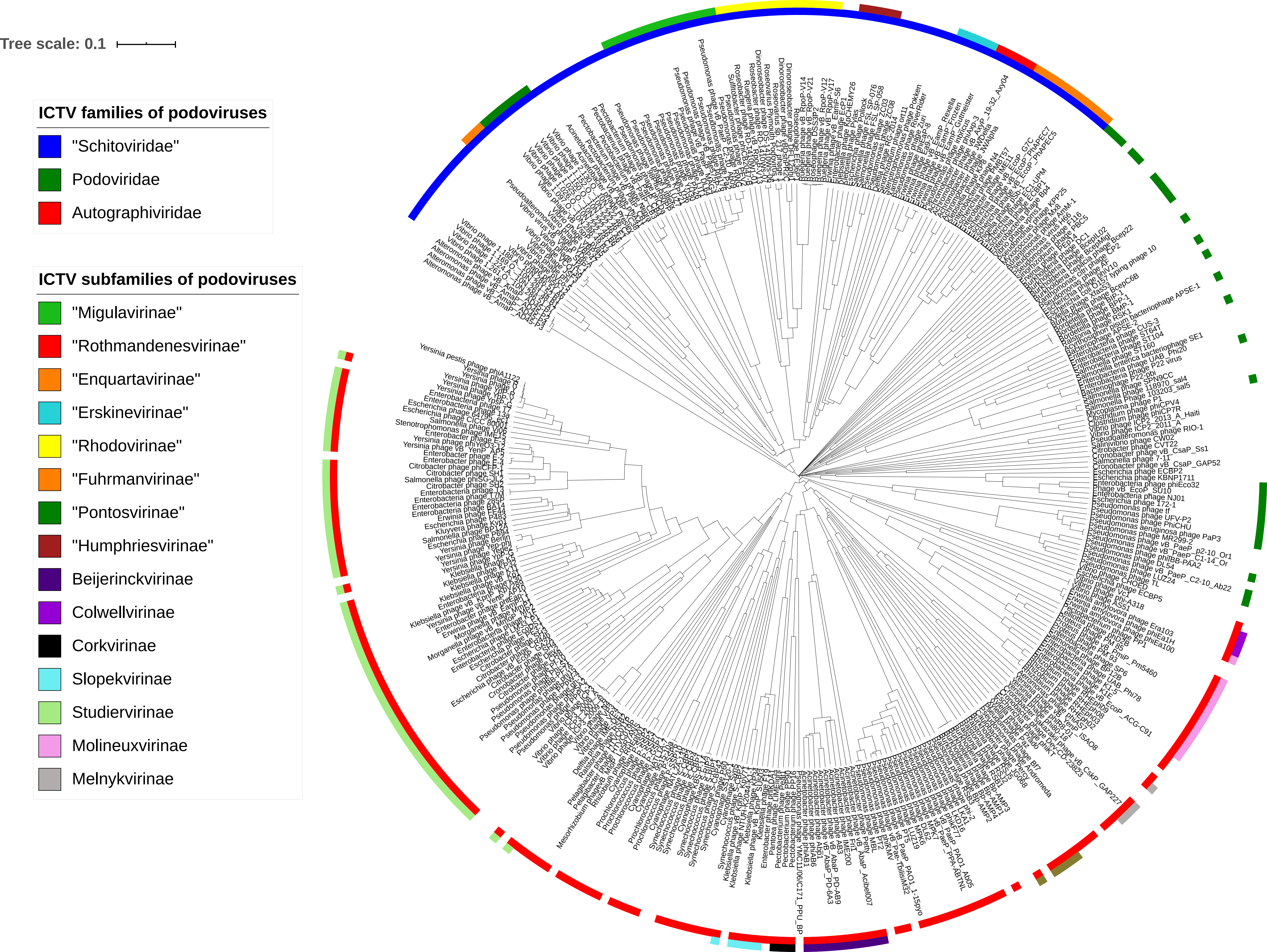
Tree scale: 0.1
ICTV families of podoviruses
"Schitoviridae"
Podoviridae
Autographiviridae
ICTV subfamilies of podoviruses
"Migulavirinae"
"Rothmandenesvirinae"
"Enquartavirinae"
"Erskinevirinae"
"Rhodovirinae"
"Fuhrmanvirinae"
"Pontosvirinae"
"Humphriesvirinae"
Beijerinckvirinae
Colwellvirinae
Corkvirinae
Slopekvirinae
Studiervirinae
Molineuxvirinae
Melnykvirinae
