# Supplementary figures and images for: From Orphan Phage to a Proposed New Family–The Diversity of N4-Like Viruses
Source: Antibiotics (Basel). 2020 Sep 30;9(10):663. doi: 10.3390/antibiotics9100663 (PMC7650795; doi:10.3390/antibiotics9100663)

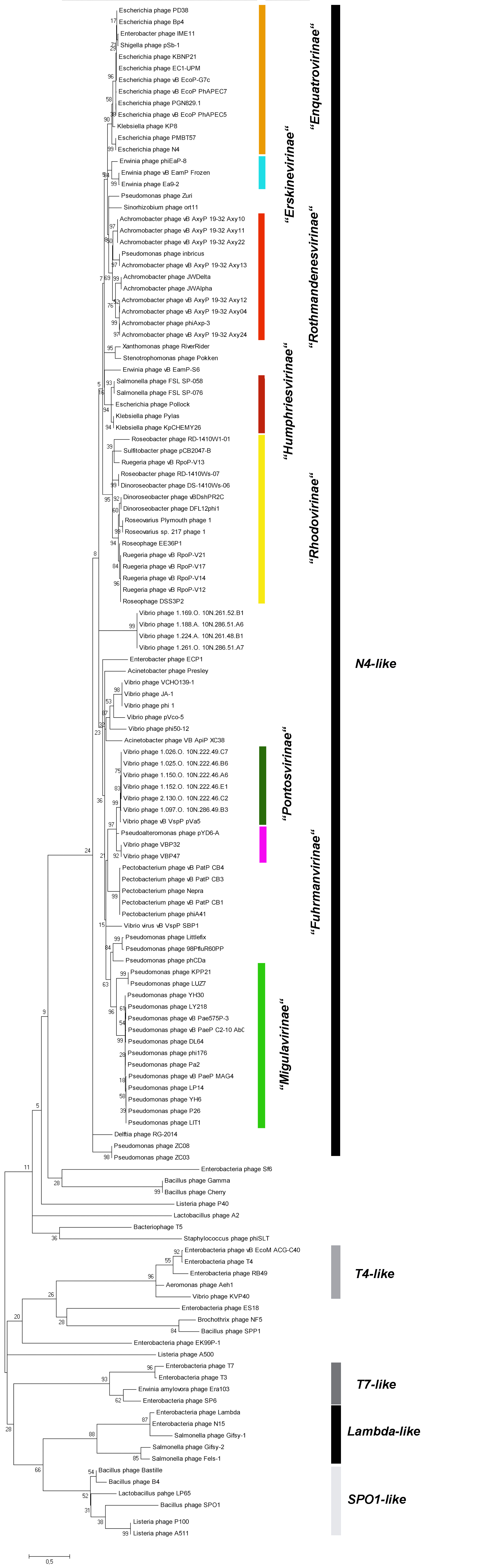

Supplement: Supplementary file 1 [file antibiotics-09-00663-s001.zip › Supplementary files/Figure S1 terminase.jpg]

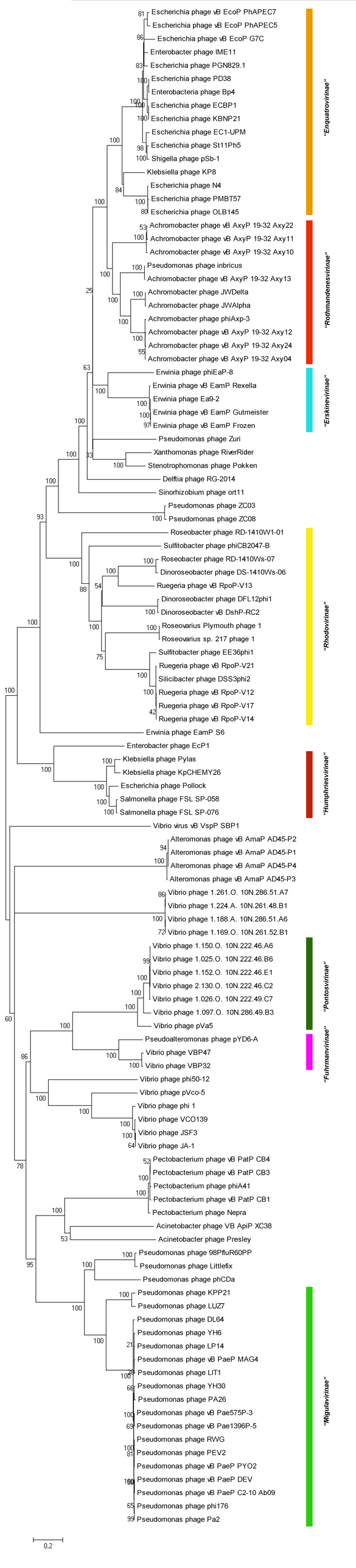

Supplement: Supplementary file 1 [file antibiotics-09-00663-s001.zip › Supplementary files/Figure S2 vRNAP.jpg]
